# Transcriptomic Analysis of the Response of Susceptible and Resistant Bitter Melon (*Momordica charantia* L.) to Powdery Mildew Infection Revealing Complex Resistance via Multiple Signaling Pathways

**DOI:** 10.3390/ijms241814262

**Published:** 2023-09-19

**Authors:** Xuanyu Chen, Kaixi Zou, Xuzhen Li, Feifan Chen, Yuyu Cheng, Shanming Li, Libo Tian, Sang Shang

**Affiliations:** 1 Sanya Institute of Breeding and Multiplication, Hainan University, Sanya 572025, China; 2 The Key Laboratory of Tropical Horticultural Crops Quality Regulation of Hainan Province, School of Tropical Agriculture and Forestry, Hainan University, Haikou 570228, China; 3 School of Life Sciences, Hainan University, Haikou 570228, China

**Keywords:** *Momordica charantia* L., *Podosphaera xanthii*, transcriptome, resistance gene

## Abstract

The challenge of mitigating the decline in both yield and fruit quality due to the intrusion of powdery mildew (PM) fungus looms as a pivotal concern in the domain of bitter melon cultivation. Yet, the intricate mechanisms that underlie resistance against this pathogen remain inscrutable for the vast majority of bitter melon variants. In this inquiry, we delve deeply into the intricate spectrum of physiological variations and transcriptomic fluctuations intrinsic to the PM-resistant strain identified as ‘04-17-4’ (R), drawing a sharp contrast with the PM-susceptible counterpart, designated as ‘25-15’ (S), throughout the encounter with the pathogenic agent *Podosphaera xanthii*. In the face of the challenge presented by *P. xanthii*, the robust cultivar displays an extraordinary capacity to prolong the initiation of the pathogen’s primary growth stage. The comprehensive exploration culminates in the discernment of 6635 and 6954 differentially expressed genes (DEGs) in R and S strains, respectively. Clarification through the lens of enrichment analyses reveals a prevalence of enriched DEGs in pathways interconnected with phenylpropanoid biosynthesis, the interaction of plants with pathogens, and the signaling of plant hormones. Significantly, in the scope of the R variant, DEGs implicated in the pathways of plant-pathogen interaction phenylpropanoid biosynthesis, encompassing components such as calcium-binding proteins, calmodulin, and phenylalanine ammonia-lyase, conspicuously exhibit an escalated tendency upon the encounter with *P. xanthii* infection. Simultaneously, the genes governing the synthesis and transduction of SA undergo a marked surge in activation, while their counterparts in the JA signaling pathway experience inhibition following infection. These observations underscore the pivotal role played by SA/JA signaling cascades in choreographing the mechanism of resistance against *P. xanthii* in the R variant. Moreover, the recognition of 40 *P. xanthii*-inducible genes, encompassing elements such as pathogenesis-related proteins, calmodulin, WRKY transcription factors, and Downy mildew resistant 6, assumes pronounced significance as they emerge as pivotal contenders in the domain of disease control. The zenith of this study harmonizes multiple analytical paradigms, thus capturing latent molecular participants and yielding seminal resources crucial for the advancement of PM-resistant bitter melon cultivars.

## 1. Introduction

Bitter melon (*Momordica charantia* L.) belongs to Cucurbitaceae, which is a mainly planted in tropical and subtropical regions, especially in Asia with an area of 340,000 ha [1], and has been widely used in the prevention and treatment of various diseases [2,3]. However, the yield of bitter melon is frequently affected by various fungal infections. Specifically, the occurrence of powdery mildew (PM) in bitter melon is attributed to the impact of fungal agents, namely *Golovinomyces cichoracearum* or *Podosphaera xanthii*, which find their niche in the cucurbit family. PM has become a primary factor responsible for the decline in bitter melon yield and occurs in all bitter melon planting areas in China, especially in the temperature ranges from 16–22 °C, where the relative humidity is increased and the light intensity is low [4]. The disease engenders necrotic lesions in the leaves and flowers, ultimately leading to the diminution of the net photosynthetic capacity. This widespread prevalence of PM, coupled with its rapid transmission and abbreviated incubation period, has incited recurrent outbreaks, thus underscoring the worldwide ramifications of this affliction [5]. In fact, the use of pesticides to control powdery mildew in the process of bitter melon planting is widespread, resulting in increased production costs and environmental pollution. Therefore, the production and use of PM-resistant bitter melon varieties is one of the most environmentally friendly, efficient, and reasonable control methods.

Conventional breeding programs have yielded a plethora of isogenic lines that exhibit varying levels of resistance to distinct PM strains. This achievement has been realized through the strategic integration of specific pathogen-resistant genes, extracted from wild resources, into cultivated varieties spanning multiple crops [6]. The intricate molecular mechanisms governing the interaction between hosts and pathogens, alongside the resultant defensive responses in pivotal agricultural species, have been dissected with a heightened degree of precision through the application of PM-resistant lines as intrinsic resources [7]. Remarkably, these lines have accrued commendation for their cost-effective attributes and environmentally -friendly nature in the context of disease management. Consequently, a compelling rationale exists to undertake an in-depth exploration into the defense mechanisms against PM in bitter melon. The primary objective is to facilitate the process of marker-assisted selection (MAS), ultimately leading to the development of elite cultivars endowed with broad-spectrum disease resistance [7,8]. In this intricate interplay between plants and pathogens, the rapid mobilization of multi-tiered defense responses emerges as a pivotal strategy to counteract infections.

Furthermore, plants display a dynamic and intricate array of responses when confronted with these challenges. The orchestration of molecular processes, including the intricate interplay of signaling molecules such as phytohormones, serves as a pivotal trigger for the activation of defense mechanisms [9,10]. Phytohormones, functioning not only as regulators of development, growth, and physiological functions, but also as conductors of defensive signals, take center stage. The harmonious and antagonistic interactions among phytohormones—encompassing abscisic acid, salicylic acid, brassinosteroids, auxins, jasmonic acid, ethylene, gibberellins, and cytokinins—constitute a complex phenomenon referred to as signaling cross-talk [11,12]. The orchestration of these phytohormones, intertwined intricately, is further nuanced by environmental cues and developmental dynamics. Salicylic acid, notably, assumes a pivotal role as a phytohormone, steering the initiation of defense mechanisms against an array of biotrophic pathogens [13,14]. The cascade of events precipitated by salicylic acid culminates in the synthesis of antimicrobial agents, including phytoalexins and pathogenesis-associated proteins. In turn, this cascade triggers hypersensitive reactions (HR) that often coincide with the programmed cell death localized in the affected region, thereby curbing the proliferation of pathogens. Contrarily, phytohormones such as jasmonic acid (JA) and ethylene (ET) are harnessed by plants to mount defenses against necrotrophic assaults, paralleling the responses initiated by physical injury [15,16].

At the crux of the matter lies the enzymatic conversion of phenylalanine into salicylic acid, a process underpinned by phenylalanine’s role as a pivotal precursor. This conversion, encompassing intermediate phases involving benzoic acid and cinnamic acid, culminates in the synthesis of salicylic acid via the shikimic acid pathway [17]. The branch of this process, facilitated by phenylalanine ammonia lyase (PAL), once assumed the exclusive mantle of salicylic acid biosynthesis. This attribution was due to the responsiveness of PAL to stress stimuli and the corresponding dip in salicylic acid accumulation triggered by PAL inhibitors [18]. The transformation of phenylalanine into cinnamic acid concurrently governs pivotal enzymes instrumental in the synthesis of fundamental lignin and flavonoids in plants. The import of the phenylpropanoid pathway is thereby underscored in the domain of plant defense [19]. Notably, the process of lignification engenders modifications in cell walls, rendering them less susceptible to enzymatic degradation, the diffusion of toxins from pathogens, and the ensnarement of pathogens. Genes linked to lignin biosynthesis are intrinsically intertwined with localized resistance against pathogens. A pertinent example lies in the transient suppression of enzymes encoded by specific genes—caffeic acid 3-O-methyltransferase (COMT), cinnamyl alcohol dehydrogenase (CAD), PAL, and caffeoyl-CoA O-methyltransferase (CCoAOMT). The attenuation of these enzymes significantly curtails wheat’s basal immunity against the fungal pathogen *Blumeria graminis* [18]. Analogous repercussions manifest in Arabidopsis and tobacco, where the suppression or absence of *PAL* genes influences the penetration resistance against both necrotrophic and biotrophic fungi. Contemporary evidence further underscores the contributions of genes associated with the lignin biosynthesis pathway, such as *ZmCCoAOMT2* in maize and *TaCAD12* in wheat. These genes confer a measurable resistance against an array of pathogens, thus illuminating the pivotal role they assume in enhancing plant defenses [20,21].

Plants, as distinct from their animal counterparts, lack the adaptive immune mechanisms characteristic of the latter. As an alternative, they have developed a multifaceted system aimed at countering invading pathogens [22]. The primary line of defense entails the recognition of pathogens by cell-surface pattern-recognition receptors (PRRs), culminating in a phenomenon termed PAMP-triggered immunity (PTI) [23,24]. This activation, encompassing receptors such as EFR and FLS2, sets into motion the MAPK signaling pathway. The resultant cascade culminates in the activation of defense genes implicated in the synthesis of antimicrobial agents [25]. In tandem, the elevation in the cytosolic calcium ion (Ca^2+^) concentration assumes a regulatory role in the generation of reactive oxygen species (ROS), in addition to orchestrating localized programmed cell death—an event commonly known as the hypersensitive response [26]. This segues into the secondary line of defense, characterized as effector-triggered immunity (ETI). Pathogens, through various mechanisms, circumvent PTI by injecting effector proteins directly into plant cells via secretion systems [23,27]. Moreover, pathogens adeptly manipulate the signaling pathways governed by plant hormones, thereby evading the host’s immune responses—a strategy typified by the coronatine toxin. Select plant species harbor specific intracellular surveillance proteins, denoted as R proteins. These proteins monitor the presence of pathogenic virulence factors, thereby triggering ETI [28]. This response entails localized programmed cell death, ultimately arresting the proliferation of pathogens and endowing cultivar-specific resistance. For instance, the overexpression of two *calcium-dependent protein kinases* (*CDPKs*) sourced from wild Chinese grapevines fortifies resistance against powdery mildew in both *Arabidopsis* and *Vitis vinifera*. Similarly, the upregulation of *TaCPK2-A* confers heightened resistance against bacterial blight in transgenic rice [29,30].

The unveiling of core pathways and the identification of responsive genes to biotic or abiotic stresses have been significantly expedited through RNA sequencing (RNA-Seq)—an avant-garde approach to probing transcriptomes. RNA-Seq facilitates a meticulous quantification of transcript levels, consequently revealing pathways primed to respond to specific stimuli. A notable instance lies in the realm of comparative transcriptome analysis, which has illuminated stress-inducible pathways and their corresponding genes in pivotal crops. This investigative avenue has extended to encompass the defensive responses of mango against *Colletotrichum gloeosporioides* [31], tomato against *Phytophthora parasitica* [32], and tobacco against *Phytophthora nicotianae* [33]. Contemporary focus has converged on transcriptomic inquiries targeting cucurbit species such as cucumber, watermelon, and melon—deciphering their inherent resilience to PM [34,35,36].

In this study, we embarked on an RNA-seq analysis for the first time, centered on the cotyledons of two distinct bitter melon strains. The first, recognized as ‘04-17-4’, exhibits resistance to PM, while the second, labeled ‘25-15’, is characterized by its susceptibility to the same. These strains were subjected to infection by *P. xanthii*, with the explicit objective of differentially expressed genes (DEGs). In our study, we delved into the intricacies of the biological processes and pathways that underpin these DEGs, which, in turn, contribute to the establishment of PM resistance. This study serves as a foundational cornerstone, intended to stimulate further explorations into the intricacies of the regulatory mechanisms that underlie PM resistance in the realm of bitter melon.

## 2. Results

### 2.1. Symptoms and Physiological Changes of Bitter Melon Leaves Infected with P. xanthii

**Figure 1:**
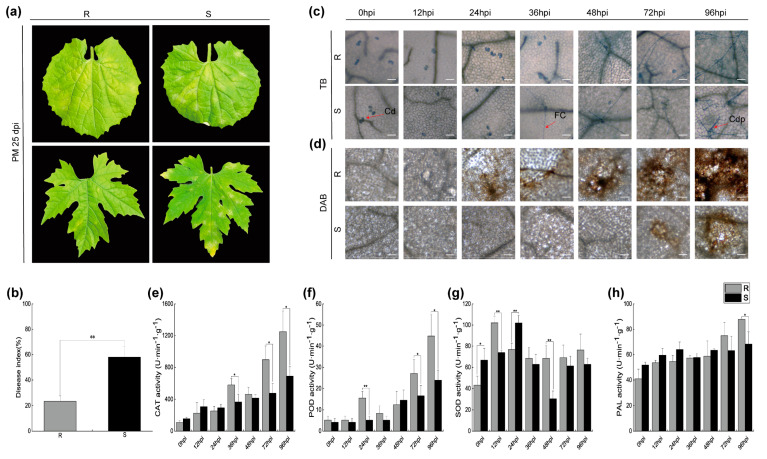
Disease symptoms in ‘04-17-4’ (R) and ‘25-15’ (S) at 0, 12, 24, 36, 48, 72, and 96 hpi with *P. xathii*. (**a**) Phenotype of the R and S varieties at 25 dpi. (**b**) Disease index assessment. (**c**,**d**) Illustration of the infection process of *P. xathii* in the R and S varieties. Cd: conidia; FC: fungal colony; Cdp: conidiophore. (**e**–**h**) Statistical analysis of the physiological indicators post-inoculation. Data represent means ± standard deviation (SD) of three biological replicates for each variety. Significance was determined using Duncan’s multiple range test, indicated by * *p* ≤ 0.05 and ** *p* ≤ 0.01.

The application of *P. xanthii* infection was executed on two distinct cultivars: the resilient ‘04-17-4’ (R) and the vulnerable ‘25-15’ (S) bitter melon leaf types. Notably, discernible symptoms of hypersensitive response emerged in the leaves of the R variant at precisely 25 days post-inoculation (dpi). The validity of this observation was further corroborated through a meticulous analysis involving the disease index (DI) (Figure 1a,b).

In order to attain a more comprehensive understanding of the dynamics underlying *P. xanthii* infection, we proceeded to introduce the pathogenic agent to the cotyledons of both the R and S cultivars of bitter melon. This was succeeded by a meticulously orchestrated series of Trypan blue and Diaminobenzidine staining procedures, implemented at varied intervals encompassing 0, 12, 24, 36, 48, 72, and 96 h post-inoculation (hpi). These rigorous observations facilitated a meticulous scrutiny of the infection progression. The resulting observations, as visually portrayed in Figure 1c, unveiled a meticulously orchestrated sequence, wherein conidiophore (Cdp) fungal colony (FC), and conidia (Cd) were successively manifested in the S cultivar at the time points of 0, 36, and 96 hpi, respectively. It is noteworthy that the spores effectively traversed their asexual growth cycle. In stark contrast, the R cultivar exhibited a distinct pattern characterized solely by the presence of spores at the 12 hpi mark. The emergence of FC materialized at the 48 hpi juncture, although Cdp remained conspicuously absent from the visual domain. This divergence is accentuated and visually captured in Figure 1d. Additionally, an in-depth scrutiny of the accumulation of ROS subsequent to the inoculation of powdery mildew unveiled a response that was both earlier and more intensified in the R cultivar in comparison with its S counterpart. This phenomenon is adeptly depicted in Figure 1d.

Concomitantly, discernible shifts in enzymatic activities surfaced during the progression of the infection process. In a comparative analysis with the S cultivar, the R cultivar distinctly showcased escalated activities across PAL, superoxide dismutase (SOD), peroxidase (POD), and catalase, all meticulously illustrated in Figure 1e–h. These findings profoundly underscore the latent potential inherent in utilizing these discrete variants of bitter melon for the explicit purpose of identifying genes that confer resistance against diseases.

### 2.2. Transcriptomic Analysis of Resistant and Susceptible Bitter Melon Leaves in Response to P. xanthii at Different Time Points

An exhaustive exploration was undertaken to investigate the transcriptomic shifts occurring on a global scale in response to the introduction of powdery mildew. This comprehensive analysis delved into the intricate leaves of both bitter melon seedlings, those displaying resistance and susceptibility. The focal point of this investigation was the interaction with the pathogen *P. xanthii*, which transpired over discrete time intervals spanning 12, 24, 36, 48, 72, and 96 hpi. The yield of this meticulous endeavor was an extraordinary accumulation of 609.59 Gb of pristine data, methodically procured from a set of 42 leaf specimens. It is worth highlighting that each of these individual samples contained no less than 5.9 Gb of data, each marked by an outstanding Q30 quality score exceeding 93.37%.

**Figure 2:**
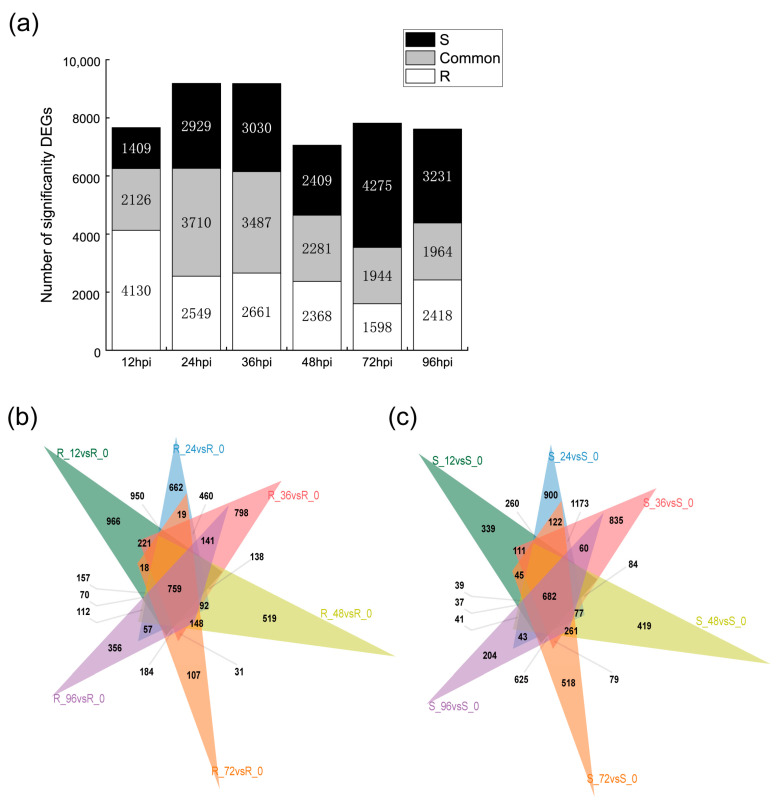
Transcriptomic overview of seven time points during *P. xathii* infection in bitter melon leaves. (**a**) Total count of transcripts significantly responsive to *P. xathii* infection. (**b**,**c**) Venn diagram depicting the number of genes affected by *P. xathii* infection over time in the R and S varieties. Genes with adjusted *p*-value (Padj) < 0.05 and |log_2_FoldChange| > 1 were considered. The numbers in the figure represent the number of DEGs for each combination.

The underpinning framework for the assembly of the transcriptome was established through a compilation of reads spanning the entirety of the samples. This foundational process is extensively detailed in Table S1. To facilitate the identification of DEGs in the leaves of both resistant and susceptible bitter melon varieties across various temporal intervals, a shrewd criterion was applied. This criterion set an adjusted threshold of *p* ≤ 0.05 and |log_2_FoldChange| > 1. This stringent approach paved the way for the recognition of gene expression differences. The process of identification was subjected to further scrutiny in a subsequent phase of analysis. As illustrated in Figure 2a, in the subset of samples demonstrating resistance and subjected to *P. xanthii* inoculation, a substantial tally of 6256 DEGs was ascertained at the 12 hpi juncture. Intriguingly, this count exhibited a gradual decline as the temporal landscape progressed, eventually stabilizing between the 36 hpi and 96 hpi periods. In stark contrast, the investigation of *P. xanthii*-infested susceptible leaves illuminated an entirely divergent trend. Here, the count of DEGs experienced an initial surge before undergoing attenuation across the entire course of the study.

In order to refine the dataset, a meticulous creation of Venn diagrams was initiated, effectively demarcating the DEGs pinpointed at distinct temporal moments 12, 24, 36, 48, 72, and 96 hpi each corresponding to the unique amalgamation of cultivar and pathogen. Spanning this extensive temporal range, an impressive assembly of 6965 DEGs was verified in the cohort of resistant samples. Remarkably, a subset of 759 DEGs demonstrated unwavering presence across all of the measured timepoints. In an analogous fashion, in the R group, a cumulative count of 6954 DEGs emerged, accompanied by a subset of 682 shared DEGs persistently maintained throughout the entirety of the investigative span (Figure 2b,c).

### 2.3. Functional Category Enrichment of Differentially Expressed Genes

**Figure 3:**
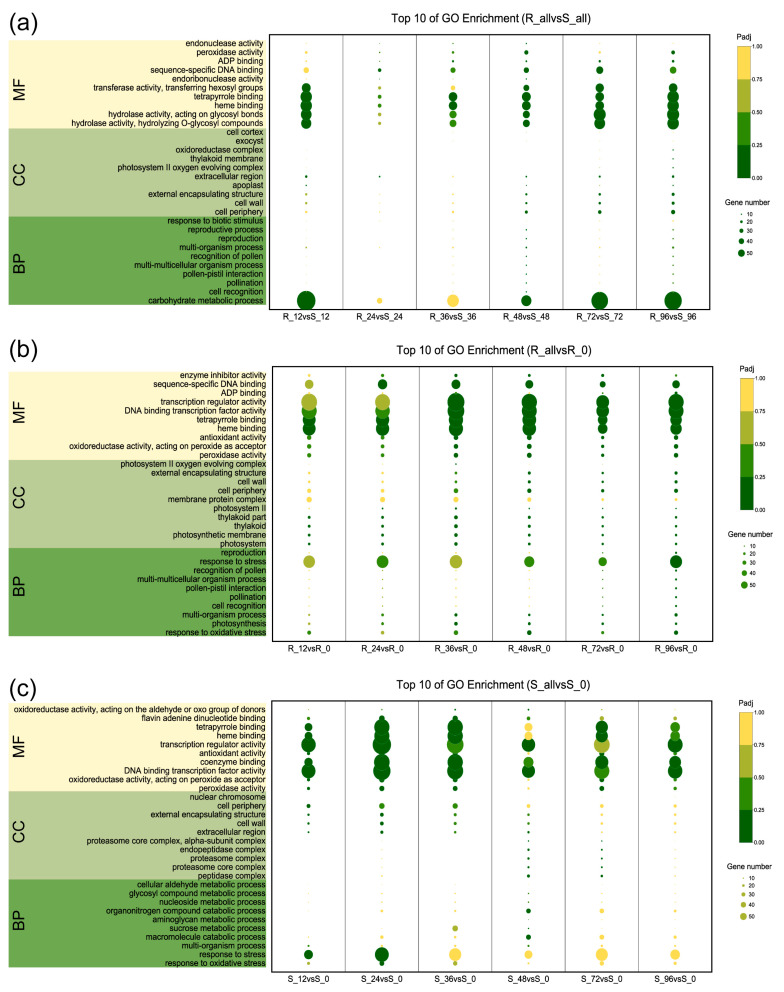
GO enrichment analysis of DEGs in bitter melon in response to *P. xathii* infection at 0, 12, 24, 36, 48, 72, and 96 hpi. (**a**) Comparison of all DEGs in R vs. S. (**b**) Comparison of DEGs in R at all time points vs. R at 0 hpi. (**c**) Comparison of DEGs in S at all time points vs. S at 0 hpi.

In an analytical pursuit, Gene Ontology (GO) and Kyoto Encyclopedia of Genes and Genomes (KEGG) analyses investigations were systematically conducted (Tables S2 and S3). The aim was to unravel the intrinsic biological mechanisms and pathways integral to the defense response against *P. xanthii* in both the resilient and vulnerable strains of bitter melon. A comprehensive breakdown of GO terminologies unveiled striking enhancements in the realm of biological processes (BP) in the resistant (R) and susceptible (S) lineages. Particularly noteworthy were the discernible enrichments in carbohydrate metabolic processes, cellular recognition, and responses to biotic stimuli when comparing R_all versus S_all. A noteworthy upswing in significance also materialized in the cellular component (CC) terms, particularly concerning the dynamics of the cell periphery and cell wall. This dynamic progression was indicative of an escalation in response to *P. xanthii* intrusion, observed more prominently in R_all versus S_all. In parallel, the molecular function (MF) terms accentuated this variance, demonstrating a substantial enrichment in R_all in contrast with S_all, notably in hydrolase activity, hydrolysis of O-glycosyl compounds, heme binding, and peroxidase activity (Figure 3a). Furthermore, with prolonged exposure to powdery mildew, the distinction in the importance of BP and CC terms deepened between R and S. This divergence was exemplified by the heightened relevance of oxidative stress responses, stress adaptation, and cell wall constituents in the resistant group (Figure 3b,c).

**Figure 4:**
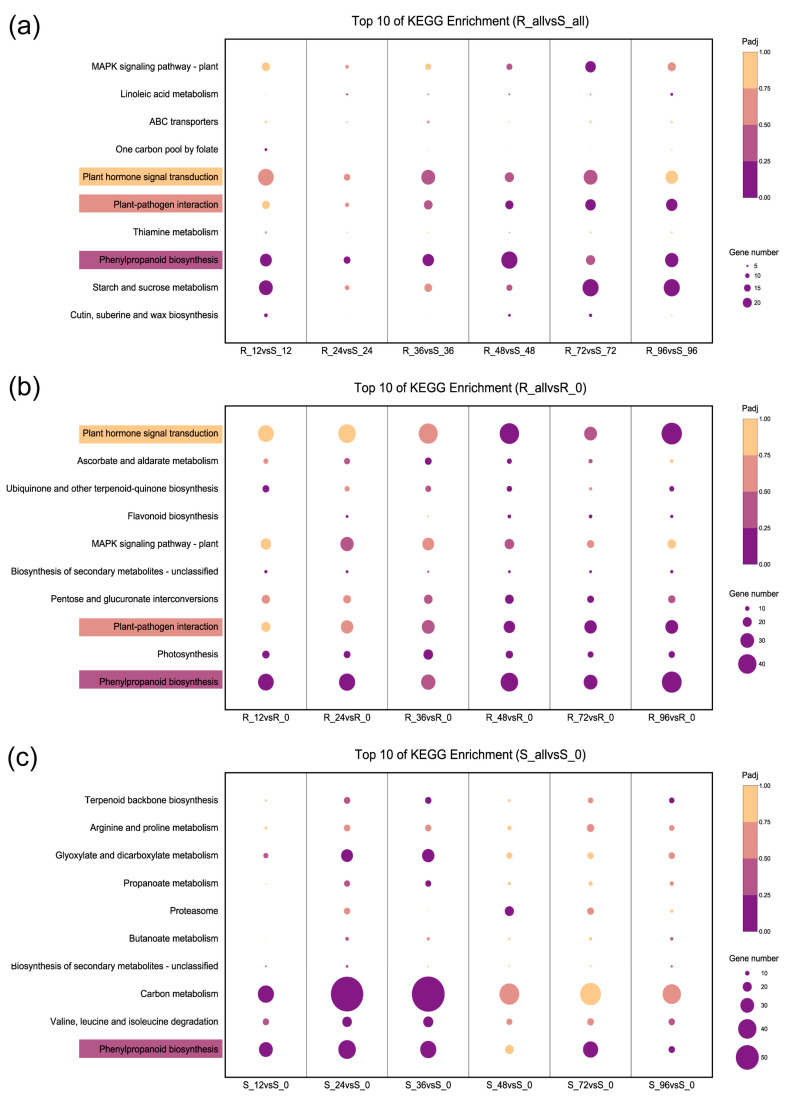
KEGG enrichment analysis of DEGs in bitter melon in response to *P.xathii* infection at 0, 12, 24, 36, 48, 72, and 96 hpi. (**a**) R_all vs. S_all. (**b**) R_all vs. R_0. (**c**) S_all vs. S_0.

Upon meticulous analysis of the KEGG classifications, a conspicuous disparity in the mechanism underpinning *P. xanthii* resistance between the robust and the susceptible lines emerged. Noteworthy contrasts were discerned in the comparison of R_all versus S_all, wherein pathways such as plant-pathogen interactions, thiamine metabolism, plant hormone signal transduction, and phenylpropanoid biosynthesis displayed significant enrichment (Figure 4a). Of particular interest was the escalating prominence of the plant hormone signal transduction and plant-pathogen interaction pathways as the duration of powdery mildew inoculation progressed in the resistant line. This phenomenon stood in stark contrast to the susceptible line, which did not exhibit a similar enrichment in the top 10 KEGG pathways (Figure 4b,c). Moreover, even though phenylpropanoid biosynthesis was a shared pathway, distinct activation patterns between R and S were evident. The resistant line exhibited a heightened mobilization of DEGs upon powdery mildew inoculation, while the susceptible line experienced a gradual decline, culminating in its lowest count at 96 hpi (Figure 4b,c). These marked deviations in metabolic pathways underscored the fundamentally diverse strategies employed by the two genotypic variants in their response to *P. xanthii* infection.

### 2.4. DEGs Related to the Phenylpropanoid Biosynthesis, SA Biosynthesis and Signaling Pathway

**Figure 5:**
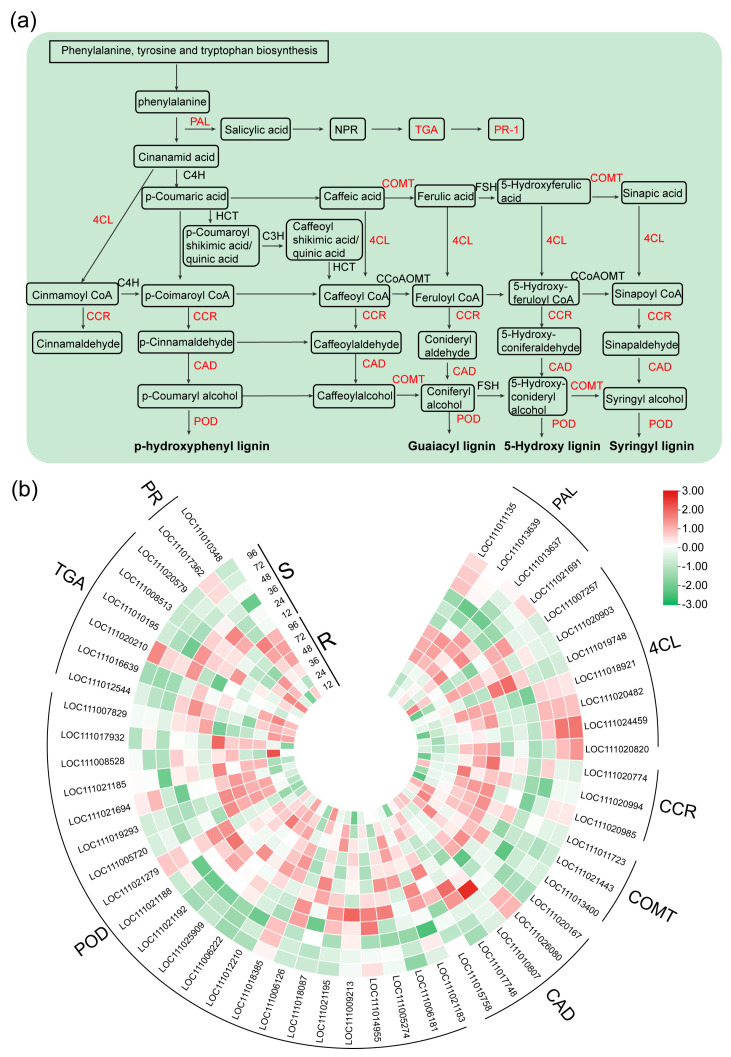
Pathways of phenylpropanoid and SA biosynthesis, SA signal transduction, and heat map of DEGs in R and S responding to infection. (**a**) Pathways of phenylpropanoid and SA biosynthesis and SA signal transduction. The red text in the figure represents the DEG families involved in regulation. (**b**) Heat map displaying log_2_FoldChange values of genes in phenylpropanoid and SA pathways in R and S relative to the control. Red indicates up-regulation and green indicates downregulation.

The results previously outlined underscore the potential regulatory function held by DEGs in orchestrating the modulation of lignin synthesis and the subsequent accrual thereof. This intricate orchestration, in turn, shapes the process of cell wall formation, a phenomenon of paramount importance due to the pivotal role played by cell walls in mediating antifungal effects. Through a more exhaustive analysis centered on the phenylpropanoid biosynthesis pathway, discernible disparities emerged in the expression profiles of various genes across the spectrum of the R and S varieties following inoculation. This observation particularly resonated in the case of genes encoding pivotal enzymes, including, but not limited to, *PAL*, *4CL*, *CCR*, *COMT*, *CAD*, and *POD* (Figure 5a,b).

Notably, the preponderance of these genes exhibited a conspicuous up-regulation in the resistant category (R) in contrast with their susceptible (S) counterparts upon encountering *P. xanthii* infection. Instances of particular note encompassed genes encoding different isoforms of (*PAL*) (*LOC111013639*, *LOC111013637*, and *LOC111021691*), varied versions of *caffeic acid o-methyltransferase* (*COMT*) (*LOC111011723*, *LOC111021443*, and *LOC111013400*), diverse forms of *cinnamoyl-CoA reductase* (*CCR*) (*LOC111020985*, *LOC111020994*, and *LOC111020774*), *CAD* (*LOC111015758*), and an array of *POD* (*LOC111019293*, *LOC111012210*, and *LOC111005720*).

Furthermore, the *PAL* genes showcased notably augmented expression levels in the resistant group (R) at 48, 72, and 96 hpi following the onset of powdery mildew infection, surpassing corresponding expression levels in the susceptible counterpart (S). Likewise, the genes encoding *CCR* (*LOC111020985*, *LOC111020994*, and *LOC111020774*), *COMT* (*LOC111011723*, *LOC111021443*, and *LOC111013400*), and *CAD* (*LOC111020167*) experienced prominent upregulation subsequent to powdery mildew infection, with their peak expression manifesting at the 96 hpi juncture in the resilient variety. It is pertinent to note that this level of induction was not mirrored by the *COMT* and *CCR* genes in response to powdery mildew infection in S.

Salicylic acid (SA), a pivotal signaling molecule, assumes a central role in orchestrating defensive responses against biotrophic pathogens. These responses entail the induction of pathogenesis-related phytoalexins, proteins, and hypersensitive responses (HR). Noteworthy in this context, three gene families associated with the SA pathway—namely PAL, TGA transcription factors, and the pathogenesis-related protein PR-1—evinced a responsive behavior to *P. xanthii* infection in both the R and S varieties (Figure 5a). Intriguingly, *PAL* genes (*LOC111013639*, *LOC111013637*, and *LOC111021691*), pivotal to SA biosynthesis, exhibited substantial upregulation in both the R and S categories. In contrast, *PR1* (*LOC111017362* and *LOC111010348*), a pivotal element of the SA signaling pathway, did not demonstrate induction and was notably downregulated in the susceptible category. Notably, it exhibited conspicuous induction in the resistant variety post-infection (Figure 5b). This underscores the robust activation of SA biosynthesis and its associated signaling pathways in R. Collectively, these outcomes underscore the significant activation of the phenylpropanoid biosynthesis, SA biosynthesis, and SA signaling pathways in response to powdery mildew infection in R, whereas these pathways remained comparably inert in S.

### 2.5. DEGs Related to the Plant-Pathogen Interaction Pathway

**Figure 6:**
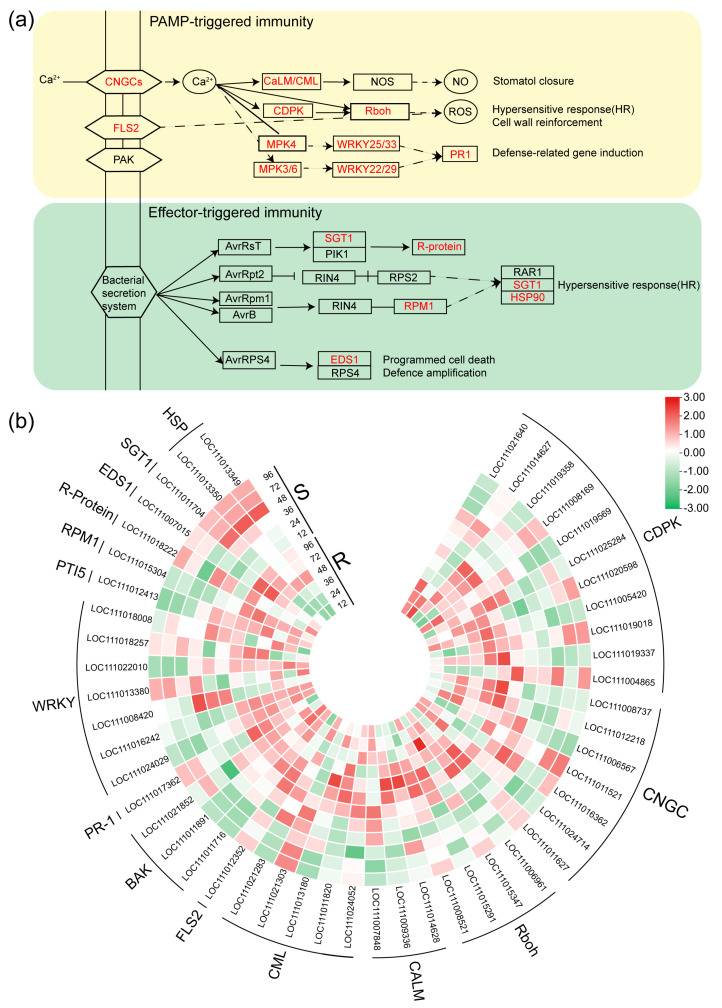
Pathways of plant-pathogen interaction and heat map of DEGs in R and S responding to infection. (**a**) Pathways of plant-pathogen interaction. The red text in the figure represents the DEG families involved in regulation. (**b**) Heat map depicting log_2_FoldChange values of genes in plant-pathogen interaction pathways in R and S, relative to the control. Red indicates upregulation and green indicates downregulation.

Following *P. xanthii* infection, a distinct subset of DEGs came to the fore, intrinsically associated with plant defense mechanisms in the bitter melon plant (Figure 6a). This particular assemblage displayed intricate intertwining with the plant-pathogen interaction pathway, and its response exhibited marked elevation subsequent to *P. xanthii* infection. Notably divergent patterns emerged between the R and S variants. Noteworthy genes implicated in *calcium-binding protein* (*CML*) (*LOC111011820*, *LOC111013180*, and *LOC111024052*), *calmodulin* (*CaM*) (*LOC111014628* and *LOC111009336*), *MAPK* (*LOC111011891* and *LOC111021852*), *serine/threonine protein kinase* (*FLS2*), and *respiratory burst oxidase* (*Rboh*) showcased conspicuous and distinct upregulation in R. This underscores the predilection of R’s defense response to *P. xanthii* to be primarily channeled through the mediation of CaM/CML-associated signal transduction. This signaling cascade, in turn, triggered the activation of the genes involved in defense responses, subsequently culminating in the generation of ROS. Conversely, S’s defense response encompassed not only signal transduction via the CaM/CML axis, but also extended to include cyclic nucleotide gated channels (CNGCs) and CDPKs. Particularly noteworthy, the genes encoding *CNGC* (*LOC111008737* and *LOC111012218*) and *CDPK* (*LOC111021640*, *LOC111019358*, and *LOC111025284*) witnessed an upregulation in R.

Furthermore, discernibly more pronounced augmentation was manifested in R in terms of defense response genes, HR, and the fortification of cell walls. This was manifested in the heightened expression of genes such as *WRKY transcription factor 22* (*WRKY22*) (*LOC111016242*), pathogenesis-related transcriptional activator genes (*PTI5*) (*LOC111012413*), and *enhanced disease susceptibility 1* (*EDS1*) (*LOC111007015*) in R. During the synthesis, these discoveries collectively pointed to the activation of the genes governing defense responses, HR, cell wall reinforcement, ROS generation, and programmed cell death specifically in R subsequent to *P. xanthii* infection (Figure 6b).

### 2.6. DEGs Related to the JA Biosynthesis and Signaling Pathway

**Figure 7:**
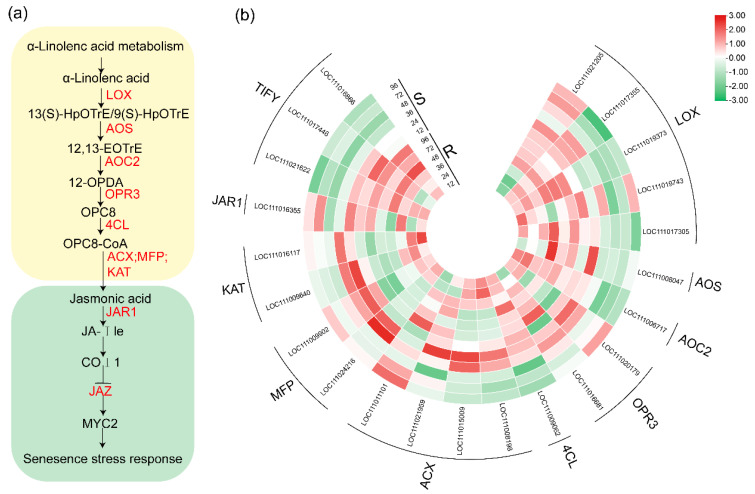
JA biosynthesis and signal transduction pathways and heat map of DEGs in R and S responding to infection. (**a**) JA biosynthesis and signal transduction pathways. The red text in the figure represents the DEG families involved in regulation. (**b**) Heat map displaying log_2_FoldChange values of genes in the JA pathway in R and S, relative to the control. Red indicates upregulation and green indicates downregulation.

The intricate biosynthesis and signaling pathway of JA yielded consequential outcomes subsequent to the incursion of *P. xanthii* (Figure 7a). Of particular significance, genes situated at the inception of the JA biosynthesis cascade displayed marked induction in the wake of infection, a response particularly pronounced in R. Among these, the *allene oxide cyclase 2* (*AOC2*) (*LOC111006717*) and *lipoxygenase* genes (*LOX*) (*LOC111019373*, *LOC111017305*) stood out. Noteworthy is the shared involvement of pivotal genes in both the JA-dependent SAR and JA biosynthesis pathways. This dual participation was manifested in a divergent pattern of expression: sustained at a restrained level in R, while undergoing induction in the S counterpart upon *P. xanthii* infection. This distinction is exemplified by the *acyl-coenzyme A oxidase* gene (*ACX*) (*LOC111998198* and *LOC111015009*), pivotal for *acyl-CoA oxidase*, as well as the *Ketoacyl-CoA thiolase peroxisomal* genes (*KAT*) (*LOC111009640* and *LOC111016117*) nestled in the JA biosynthesis pathway. Furthermore, the JA signaling pathway demonstrated a pronounced interplay of regulatory elements. In this intricate network, the negative regulatory factors typified by *Jasmonate ZIM-domains* (*JAZs*) (*LOC111021622*, *LOC111017446*, and *LOC111016886*) underwent substantial induction in R. In stark contrast, these elements remained inert in S after *P. xanthii* infection (Figure 7b).

### 2.7. Identification of Key Hub Genes and Network Construction

**Figure 8:**
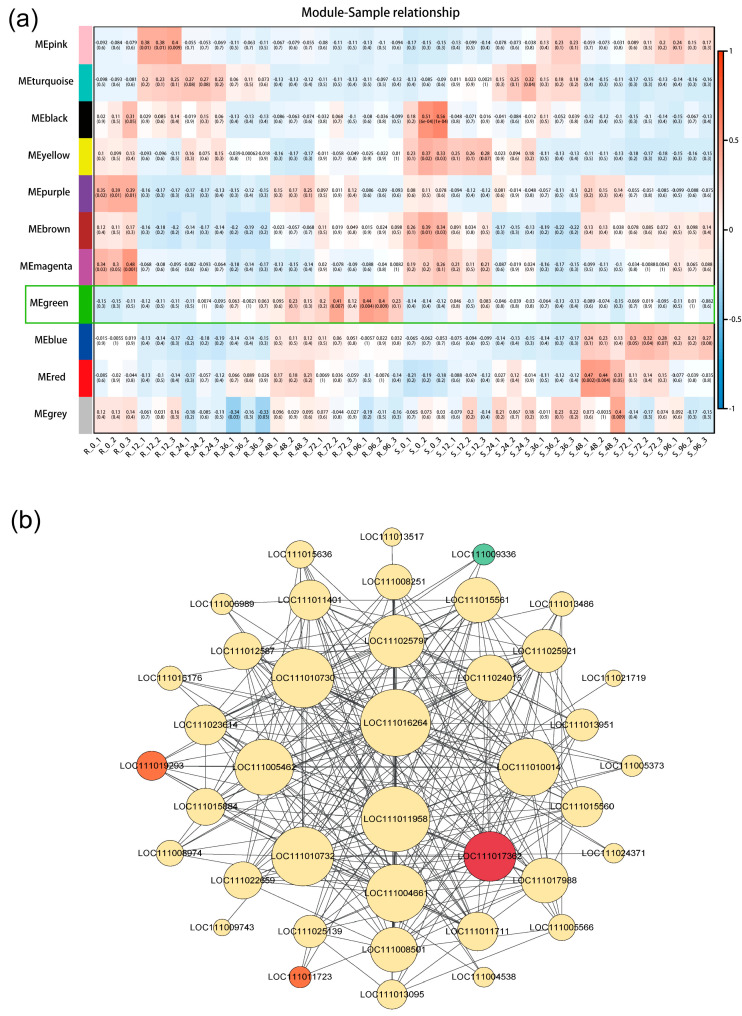
WGCNA of key modules associated with *P. xanthii* defense in bitter melon. (**a**) Gene expression profile in each module. (**b**) Network representation of the green module relationships. Red, green, and orange represent *PR1*, *CALM*, and *POD* genes, respectively.

In the pursuit of identifying the pivotal genes associated with *P. xanthii*-induced stress, we conducted an analysis of the substantial pathways delineated by the DEGs implicated in this intricate process. Employing stringent criteria encompassing MM > 0.75 and an edge weight value surpassing 0.30, we unearthed notable insights. Remarkably, in the verdant module, DEGs in the R strain exhibited a robust expression, while their counterparts in the S strain displayed an antithetical pattern (Figure 8a). It is pertinent to note that the genes in the green modules were systematically ranked according to their co-expression connectivity. Subsequently, we selected the uppermost 10% of genes with the highest connectivity for detailed analysis. This cohort encompassed genes of significance, including WRKY transcription factors (*LOC111024015*, *LOC111015884*, *LOC111015884*, and *LOC 111004661*), peroxidase (*LOC111017988* and *LOC111019293*), and pathogenesis-related proteins (*LOC111013095*, *LOC111013517*, and *LOC111017362*). Additionally, PR-1 (*LOC111017363*), COMT (*LOC111011723*), CML (*LOC111009336*), and peroxidase (*LOC111019293*) demonstrated prominence, underpinned by their enrichment in the KEGG pathways (Figure 5b and Figure 6b). The functional annotation of these genes unequivocally underscored their alignment with defense responses, phenylpropanoid biosynthesis, plant-pathogen interaction, and the intricate web of plant hormone signal transduction.

### 2.8. Verification of DEGs Using Quantitative Reverse-Transcription PCR (qRT-PCR)

**Figure 9:**
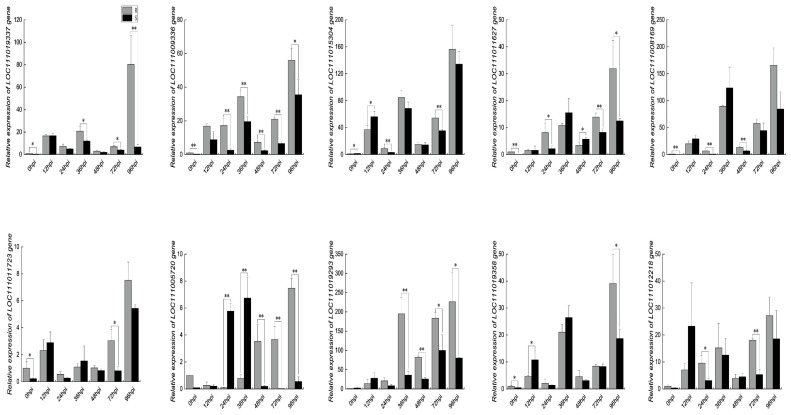
Expression analysis of selected DEGs at 0, 12, 24, 36, 48, 72, and 96 hpi using the 2^−ΔΔCt^ method. Data represent means ± SD of three biological replicates for each variety. Significance was determined using Duncan’s multiple range test, indicated by * *p* ≤ 0.05 and ** *p* ≤ 0.01.

In order to establish the robustness of the RNA-seq data and to conduct a thorough examination of the expression patterns exhibited by stress-responsive genes, a meticulously curated set of 10 DEGs underwent comprehensive qRT-PCR assays. These meticulously designed experiments spanned seven discrete time points (0, 12, 24, 36, 48, 72, and 96 hpi) and encompassed both the R and S strains (Figure 9). Strikingly, the expression profiles of the majority of DEGs consistently mirrored the outcomes derived from the RNA-seq analysis, standing as a resounding confirmation of the reproducibility of the data. Notably, it is imperative to highlight that the dynamics of gene expression experienced more pronounced fluctuations in the R strain as opposed to the S strain, following the incursion of *P. xanthii* infection.

## 3. Discussion

This comprehensive study was meticulously devised with the aim of unraveling essential genomic factors pertinent to the resilience of bitter melon (*Momordica charantia* L.) against the pathogenic agent *P. xanthii*. It merits attention that the pathogen *P. xanthii* does not engage in the direct infiltration of bitter melon fruit. Rather, its deleterious impact is exerted by targeting the vegetative vigor, thereby adversely affecting fruit yield, through its intrusion into the leaves of the bitter melon. This incursion induces a decline in photosynthetic efficiency, coupled with an elevation in the rates of respiration and transpiration. As a natural consequence, the identification of resistance-associated genes post-infection emerges as an imperative measure to alleviate the detrimental impact on yield. Thus, with this specific objective at the forefront, a judicious selection of time points was undertaken—0, 12, 24, 36, 48, 72, and 96 hpi—to facilitate the subsequent RNA-seq analysis. This decision was grounded in the distinct temporal completion of the asexual spore growth cycle observed in both the resistant and susceptible lines of bitter melon. In the contextual domain of this study, it remains salient that the resistant lineage of bitter melon exhibited a conspicuously augmented abundance of DEGs intricately connected to the interactions between plants and pathogens, biosynthesis of lignin, and the biosynthesis and signaling cascades of SA and JA. This augmentation was in stark contrast with the susceptible line, which showed a subdued expression of these genes when subjected to the onslaught of *P. xanthii*. It is noteworthy that this finding resonates with previous transcriptomic explorations conducted with cucumber, watermelon, and tobacco. These antecedent investigations similarly unveiled that resistant strains tend to manifest an enhanced DEG expression profile when juxtaposed with their susceptible counterparts during the ordeal of biotic stress [33,34,36].

Significantly, the intricate orchestration of plant defense mechanisms in response to biotic stress is underpinned by hormone-mediated signaling pathways. In particular, the signaling conduits orchestrated by JA and ET substantively contribute to the establishment of plant resistance against necrotrophic pathogens, while the SA-mediated pathway assumes a pivotal role in fortifying plant defenses against biotrophic pathogens [37]. In the ambit of our experimental framework, it is imperative to accentuate that the triad of genes encoding *JAZs* (*LOC111021622*, *LOC111017446*, and *LOC111016886*) evinced a heightened state of expression exclusively in the context of the resistant bitter melon subsequent to the incursion by *P. xanthii*. The JAZ protein, being a pivotal component in the transduction of JA signals, functions as a negative modulator that directly obstructs the initiation of JA-responsive transcription factors [38]. Furthermore, in the spectrum of our inquiry, it is of momentous significance to highlight the upregulation of three genes implicated in the biosynthesis of SA (*LOC111013639*, *LOC111013637*, and *LOC111021691*) as well as the elevation in expression of two genes encoding pathogenesis-related proteins (*PR-1*; *LOC111017362* and *LOC111010348*) in the resistant line as compared to its susceptible counterpart. It is of paramount importance to acknowledge that PR genes are pivotal constituents orchestrating plant defense against pathogens, with their prominence underscored by a well-established body of literature [39,40,41]. In this study, PR1 was highly expressed in R variety. Similar studies have shown that overexpression of PR1 gene can enhance the resistance of melon to PM and FOM [42]. This tenet is fortified by empirical evidence showcasing that transgenic tobacco plants, characterized by an overexpression of the PR-1 gene, demonstrated heightened resistance to *P. parasitica*. Conversely, plants in which the PR-1 gene was silenced displayed an elevated susceptibility. Clearly, PR-1 stands poised as a constructive regulator of plant resilience against *P. parasitica*. It is both pertinent and cogent to deduce that the augmentation in systemic acquired resistance (SAR), aligned with the intensified expression of PR-1, is intrinsically interwoven with defense mechanisms predicated upon SA [43,44]. This alignment with the SA pathway serves to underscore the pivotal role played by the induced systemic resistance and SAR triggered by JA/SA in engendering a distinct and robust line of defense against the encroachment of *P. xanthii* in the realm of bitter melon.

This research was executed with an exceptional degree of precision, aimed at unraveling the intrinsic genomic factors that underscore the resistance mechanisms in bitter melon (*Momordica charantia* L.) against the pathogenic intruder *P. xanthii*. A salient observation emerges: *P. xanthii* avoids direct infiltration into bitter melon fruit, opting instead to target and compromise the vigor of its vegetative faculties and the yield of its fruits by directing its attack towards the bitter melon leaves. This intrusion eventuates in a compromised photosynthetic process, accompanied by a concomitant escalation in respiration and transpiration rates. The identification of genes associated with resistance, in the wake of infection, emerges as a pivotal strategy to counteract yield depreciation. Hence, a judicious selection of time intervals, encompassing 0, 12, 24, 36, 48, 72, and 96 hpi, was undertaken for the purpose of conducting an incisive RNA-seq analysis. This deliberate selection finds validation in the discrete culmination of the asexual spore growth cycle evident across both of the strains of bitter melon, whether resistant or susceptible. A prominent facet of this study rests on the conspicuous augmentation of general phenylpropanoid pathways and the pathways specifically related to lignin. These pathways have proven themselves as pivotal regulators of defense mechanisms against pathogenic incursions in a diverse spectrum of plant species [45,46]. The prevailing literature accentuates the robust and pervasive response of phenylpropanoid biosynthesis genes across a gamut of plant taxa when confronted by pathogenic exigencies [47,48,49]. The correlation between the combat against white rust disease in chrysanthemum, the fortification against early blight in tomatoes, resistance to leaf blight in rice, and resistance to Bgt in Tibetan barley emerges, linking these instances to the mechanisms inherent to the phenylpropanoid pathways. It is imperative to underscore that the spectrum of phenylpropanoids encompasses an array of compounds, inclusive of lignin, flavonoids, and phenolic compounds, emerging as the outcome of distinct downstream pathways [25]. A meticulous scrutiny of the DEGs affiliated with the phenylpropanoid pathway and the biosynthesis of monolignols uncovers the induction of multiple genes across both the resistant and susceptible lines subsequent to PM infection (Figure 5a,b). This harmonizes with an array of sources that highlight the induction of genes involved in the coding of pivotal monolignol biosynthesis proteins post pathogenic encounters across a plethora of plant species. A precedent set by a wheat study elucidates the accumulation of transcripts from monolignol genes in the resistant strains upon PM infection [18,50,51]. This pattern echoes in instances such as watermelon powdery mildew interactions, where there is a conspicuous upregulation of genes related to defense mechanisms [36]. Our research findings shed light on the amplified expression of *PAL* genes in the R_all vs. R_0 comparison. This resonates with prior discoveries where heightened mRNA transcripts of the PAL enzyme were documented in barley subsequent to PM infection [52]. Amidst this complex array of responses, genes of the *4-coumaroyl-CoA ligase* (*4CL*) lineage play a cardinal role in orchestrating CoA ester production, thereby assuming multifarious functions during times of plant stress [53,54]. However, our findings diverge, pointing towards the escalated expression of *4CL* genes in the susceptible line in contrast with the resistant line. This discrepancy might well be attributed to divergent upstream metabolite production. The purview of *O-methyltransferase* (*OMT*) genes, encompassing *COMT* and *CCoAOMT*, encompasses multiple branches of the phenylpropanoid pathway. These genes enhance the defense potential against pathogens through the methylation of secondary metabolites, such as alkaloids, phenylpropanoids, and flavonoids [55]. For instance, the wheat *TaCOMT-3D* gene signifies the resilience against eyespot disease. In our investigation, specific *COMT* genes exhibited induced expression in the resistant line in comparison with the susceptible line (Figure 5a,b), accentuating the plausible affirmative role enacted by our OMT family isoforms in fortifying resistance mechanisms. Under the aegis of PM-induced stress, supplementary genes, such as *cinnamoyl CoA* reductase (*CCR*), emerge as contributors to the resistance profile of ‘04-17-4’. Earlier studies underscore the role of *CCR* genes in augmenting the accumulation of monolignols, the foundational constituents of cell walls [56]. Additional validation stems from instances such as the induction of Arabidopsis *AtCCR2* by the pathogenic agent *Xanthomonas campestris* pv. campestris, underscoring its significant role in plant defense. Correspondingly, the *OsCCR1* gene in rice, upon induction, plays a part in the defense mechanism against *Xanthomonas oryza* [57]. In a parallel vein, *CCR* genes have been implicated in the realm of UV stress resistance. Amidst this labyrinth of intricate defense mechanisms, peroxidases (EC1.11.1.7) emerge as pivotal inducible proteins post-inoculation [58]. Constituting a comprehensive multigene family, these peroxidases shoulder critical roles in diverse activities, including cell wall cross-linking, suberin synthesis, lignin formation, and activities tied to RNS and ROS, not to mention their pivotal role in hypersensitive response in resistant plants [59,60]. A salient facet materializes through the role played by *PRX* genes in the oxidation of monolignol units, subsequently culminating in lignin polymerization [61]. A trail of evidence points to the augmentation of POD activities post-infection in plants. The expression of *PRX* genes, for example, is induced by Bgt attack in the context of wheat mesophyll and epidermal tissues [62]. Likewise, there is a documented surge in peroxidase activities in *Pichia galeiformis*-infected citrus plants [63]. Recent research in watermelon and melon underscores the induction of a plethora of peroxidase-encoding genes in resistant lines, while their counterparts in susceptible lines maintain a neutral stance following PM infection [36,64]. Interestingly, this investigation reveals the identification of a total of 22 DEGs that differentiate between resistant and susceptible lines, in which there is a remarkable upregulation of the majority of *PRX* genes in the resilient category. These findings resonate with the escalated POD activity observed in cotton in response to *Verticillium dahlia* infection [65]. The marked induction of *PRX* genes in resistant lines underscores their potential contribution to the defense arsenal against PM infection.

Throughout the course of their extensive coevolution with pathogenic microorganisms, plant-pathogen interactions have led to the development of unique defense mechanisms in plants. The natural immune system of plants is commonly categorized into two tiers. The initial tier, recognized as pathogen associated molecular pattern (PAMP) triggered immunity (PTI), hinges on the identification of diverse pathogens and microbes by pattern recognition receptors positioned on the surfaces of plant cell membranes. Consequently, PAMPs initiate PTI responses. The subsequent tier of immune response is fundamentally based on the evolutionary emergence of resistance genes in plants, allowing them to indirectly or directly perceive pathogens and subsequently release corresponding effectors to initiate ETI responses [23]. Chitin stands as one of the few well-established PAMPs. When plants come into contact with pathogens, pattern recognition receptors on their cell membranes differentiate chitin and chitin oligosaccharides on the pathogens, instigating a cascade of responses [66]. In the context of this study, we identified genes displaying a differential expression and engaged in the regulation of the Ca^2+^ signaling pathway in response to *P. xanthii* infection (Figure 6a,b). These genes encompass *CDPKs*, *CaM/CML*, and *CNGCs*. CNGCs constitute a family of ligand-gated calcium channels in plants, and research has corroborated their role in the defense response to pathogens [67]. Dysfunction in CNGCs disrupts the crucial Ca^2+^ signaling pathway governing plant defense mechanisms [68]. We observed the downregulation of specific *CNGC* genes at 36 hpi in the susceptible samples, whereas they exhibited upregulation at 48 hpi in the resistant samples. CaM/CML proteins operate as pivotal sensors in the transduction of Ca^2+^ signals. Prior investigations have highlighted that irregularities in CaM/CML expression and functional mutations significantly influence immune responses [24,69]. Our analysis of plant-pathogen interactions uncovers more transcriptional modifications in *CaM/CML* genes in the resistant samples following *P. xanthii* infection (Figure 6a,b). CDPKs, essential receptors in Ca^2+^ signal transduction [70], assume multifaceted roles in diverse physiological processes, encompassing the regulation of biotic stress [71,72]. In our current study, we documented a greater number of differentially expressed *CDPK* genes at 36 hpi in the susceptible material compared with the resistant material, characterized by a predominant downregulated trend (Figure 6a,b). These findings suggest that the calcium signaling pathway holds the potential to serve as a positive regulator of PM resistance. The extensive WRKY transcription factor family, comprised of numerous plant transcription factors, assumes both positive and negative regulatory roles in plant defense responses [73]. An antecedent investigation showcased that upon tomato infection by *O. neolycopersici*, the WRKY transcription factor *ShWRKY81* underwent robust and rapid induction, and its overexpression substantially elevated resistance to *O. neolycopersici* [74]. In our ongoing study, we have identified seven differentially expressed WRKY transcription factors, with four consistently upregulated in the resistant material. Intriguingly, the expression trends of these genes in the susceptible material contrasted with those in the resistant material. Our findings were in accordance with the results of recent studies, in that WRKY TFs are involved in the defense of Cucumis sativus to PM [75]. This implies that fluctuations in the expression of pivotal transcription factors may underlie the divergent resistance mechanisms exhibited by bitter melon against *P. xanthii*. These revelations furnish novel insights into the potential molecular mechanisms that underlie the resistance of bitter melon to *P. xanthii*. Furthermore, ETI signaling pathways play a pivotal role in the plant’s responses to biotic stress. Our investigation has unveiled substantial upregulation of 1 *PTI5*, 1 *RIN4*, and 1 *EDS1* in the R variety compared with the S variety post-inoculation, suggesting distinct mechanisms and functions of these genes in discrete pathways linked to disease resistance.

## 4. Materials and Methods

### 4.1. Plant Materials and P. xanthii Inoculation

The bitter melon specimens utilized in this study encompassed two distinct lines: the PM-resistant strain ‘04-17-4’ (R) and the PM-susceptible strain ‘25-15’ (S), both of which were provided by Hainan University. The germinated seeds were planted in a nutrient-rich soil mixture comprising peat, perlite, and fertilizer, blended in a ratio of 6:3:1. These were then placed in a growth chamber programmed for a light period of 14 h at 26 °C, followed by a dark period of 10 h at 22 °C, maintaining a relative humidity of 60%. Upon reaching the four-leaf growth stage, the bitter melon seedlings underwent *P. xanthii* inoculation. This involved the application of the pathogen, with its concentration adjusted to 1 × 10^6^ conidia/mL and mixed with 1% (*V*/*V*) Tween 20 [35], onto the first and second fully expanded leaves. These leaves, enumerated from the uppermost part of the bitter melon seedlings, were subjected to the inoculation process through spraying. Subsequent to inoculation, leaf samples were collected at seven distinct time points: 0, 12, 24, 36, 48, 72, and 96 hpi. Each treatment condition was subjected to three independent experimental runs. Following collection, the leaves from all of the experimental groups were promptly sampled and stored at a temperature of −80 °C.

### 4.2. Investigation of Staining and Disease Index

Trypan blue staining was employed to discern the alterations induced by *P. xanthii* in the inoculated bitter melon cotyledons. The in -situ accumulation of hydrogen peroxide (H_2_O_2_) was determined via meticulous histochemical analysis. The detection of H_2_O_2_ followed the 3,3′-diaminobenzidine uptake methodology, as outlined in the literature [76]. Distinct assessments of Fusarium wilt resistance were conducted at an interval of 25 dpi. Each seedling underwent individual phenotypic classification, wherein severity ratios (0 = no or almost no symptom; 1 = faint spot and below the 5% of leaves; 3 = thin mat of mildew and 6–10% of the leaves; 5 = thick mat of mildew and 11–20% of the leaves; 7 = very thick mat of mildew and 21–40% of the leaves; 9 = whole leaf surface coated with mildew and above 40% of the leaves) were assigned based on established criteria [77]. To quantify the resistance, a disease index (DI) was formulated using the following equation: DI = Σ (number of diseased plants × representative value of this level)/(total number of plants × representative value of the highest level) × 100.

### 4.3. Determination of Enzymes Activity

Enzyme activities comprising CAT, POD, SOD, and PAL were evaluated at seven discrete time intervals, encompassing both the ‘04-17-4’ (R) and ‘25-15’ (S) strains. This evaluation adhered to the protocols stipulated in the Experimental Course of Plant Physiology. To uphold precision, each test sample was subjected to triplicate repetition. Consequently, the magnitudes of CAT, POD, SOD, and PAL activities were gauged in line with the furnished guidelines and quantified through the employment of a T6-1650E UV spectrophotometer.

### 4.4. RNA Isolation and Sequencing

The extraction of the total RNA from the samples was conducted using the TRIzol reagent, in accordance with the procedural guidelines specified in the kit instructions. Following the extraction process, a comprehensive evaluation of the RNA samples’ quality was conducted using Nanodrop LITE (Thermo, MA, USA) and Agilent 2100 (Agilent, CA, USA), and the RIN of the samples is included in Table S4. Subsequent to this, the RNA samples were transmitted to Novogene (Beijing, China) for RNA sequencing via the Illumina Novaseq 6000 platform, incorporating 150-bp paired-end reads. This sequencing procedure strictly adhered to the prescribed protocols established by the manufacturer. Notably, the encompassed steps encompassed the construction of cDNA libraries and Illumina sequencing. It is worth highlighting that each sample underwent a repetition of the sequencing process three times, a practice that enhanced the robustness and reliability of the experimental methodology.

### 4.5. RNA-Seq Data Analysis

Through meticulous filtration of the raw data, a dataset of high quality was achieved by effectively removing joint and low-quality reads. Subsequently, the refined data underwent alignment to the bitter melon genome database1, employing HISAT2 v2.0.5. Successful alignment was defined by disparities of no more than 2 mismatches between the default reads and the reference genome sequences. The quantification of read counts assigned to individual genes was accomplished using the Feature Counts tool (1.5.0-p3), establishing these counts as the foundational expression levels for the respective genes. Further normalization was conducted by converting the expression values into fragments per kilobase million (FPKM). To perform differential analysis of the gene expression, DESeq2 (1.16.1) was utilized, leading to the identification of DEGs based on specific criteria: |log_2_FoldChange| > 1 and adjusted *p*-value < 0.05. In pursuit of an exhaustive assessment of gene functionalities, all DEGs underwent systematic mapping, utilizing the resources of the GO and KEGG databases. The threshold to determine significant enrichment was established at an adjusted *p*-value < 0.05.

### 4.6. Construction of the Weighted Gene Co-Expression Network

The creation of co-expression networks was undertaken by employing the Weighted Gene Co-expression Network Analysis (WGCNA) on the Metware Cloud platform (https://cloud.metware.cn/ (accessed on 9 July 2023)). Commencing with the filtration of genes, the ensuing expression values were imported into the WGCNA package to initiate the construction of co-expression modules. Subsequent to this, the computation of expression correlation coefficients was executed among the remaining genes, thus facilitating the determination of an optimal soft threshold. This threshold was pivotal for the development of gene networks, which adhered to a scale-free topology model [78].

To uncover biologically significant modules, the eigenvalues of the modules were harnessed to calculate correlation coefficients. The assessment of intramodular connectivity, recognized as the soft connectivity function, was applied to each gene. Remarkably, genes demonstrating the highest connectivity, often within the top 1% or 5%, were singled out as hub genes. The graphical depiction of these intricate networks was achieved by employing Cytoscape version 3.5.1.

### 4.7. qRT-PCR Validation

A cohort of ten genes, exhibiting a spectrum of distinct expression patterns, ascertained through the RNA sequencing process, was purposefully selected for validation via quantitative real-time reverse transcription PCR (qRT-PCR). The process of RNA extraction from the samples engaged in the RNA-seq experiment was meticulously undertaken employing the Plant Total RNA Isolation Kit (FOREGENE, Chengdu, China). The synthesis of first-strand cDNA was facilitated using the MonScript™ RTIII All-in-One Mix with dsDNase (Monad, Suzhou, China). To formulate primers specifically tailored for qRT-PCR, the genetic blueprints were harnessed from the corresponding sequences available on the NCBI platform, and these primer specifications are comprehensively detailed in Table S5. To establish a stable internal reference, *McActin7* was employed. The ensuing qPCR procedures were executed using the ChamQ Universal SYBR qPCR Master Mix (Vazyme, Nanjing, China), seamlessly integrated with the Applied Biosystems QuantStudio 1 instrument (ABI, MA, USA). The procedural orchestration closely adhered to the manufacturer’s prescribed protocols. To ensure precision, each gene underwent rigorous analysis involving three technical replicates. The determination of the relative gene expression levels was rooted in the 2^−ΔΔCt^ methodology, culminating in a graphical presentation of the results. An unwavering commitment to rigorous methodology was upheld, with a minimum of three independent experiments conducted for each sample. Remarkably, each experimental iteration featured three technical replicates, fortifying the robustness, dependability, and homogeneity of the outcomes.

### 4.8. Data Analysis

The utilization of plant materials and the meticulous preparation of the *P. xanthii* inoculum formed indispensable facets of this study. The entirety of the numerical data garnered from the research were subjected to rigorous statistical analysis and subsequent assessments of significance. To elucidate further, the physiological data procured subsequent to *P. xanthii* infection underwent meticulous examination via the medium of one-way analysis of variance (ANOVA), with the determination of statistical significance being contingent upon pairwise comparisons (*p* < 0.05). This analytical protocol was executed utilizing SPSS 23.0 (Statistical Package for the Social Sciences, Chicago, IL, USA). The depiction of expression patterns in the realm of DEGs was realized by graphically representing the log_2_FoldChange values. This visualization was achieved through the generation of a heatmap, facilitated by both the TBtools v1.120 (China) and Originpro 2021 (USA) software platforms.

## 5. Conclusions

This study marks a pioneering contribution by unraveling the intricate molecular mechanisms underpinning bitter melon resistance. Employing RNA-seq, we have presented the genetic landscape governing plant resistance to PM, heralding a novel perspective in this domain. The elucidation of multiple regulatory pathways underscores their pivotal role in conferring resistance to fungal pathogens in bitter melon. Specifically, the orchestration of lignin synthesis, as well as SA/JA biosynthesis and signaling, coupled with the mediation of calcium ion-triggered MAPK pathways, emerges as closely intertwined with the innate disease resistance of this plant. The integration of WGCNA alongside the scrutiny of hub gene networks has shed light on the key regulatory factors triggered in response to PM infection. The utilization of the two bitter melon varieties proves strategic, not only serving as apt materials for hybrid population construction, but also offering an invaluable resource for the subsequent mapping of resistance-related genes. In essence, these findings not only serve as a stepping stone for future research on bitter melon resistance, but also lay the groundwork for the strategic development of bitter melon varieties boasting fungal resistance or even durable, broad-spectrum resistance.

## Data Availability

The raw data from the transcriptome analysis are deposited in the SRA database under accession number PRJNA1007942.

## Supplementary Materials

The following supporting information can be downloaded at: https://www.mdpi.com/article/10.3390/ijms241814262/s1.

## References

[1] Dhillon N.P.S., Sanguansil S., Srimat S., Laenoi S., Schafleitner R., Pitrat M., McCreight J.D. (2019). Inheritance of Resistance to Cucurbit Powdery Mildew in Bitter Gourd. HortScience.

[2] Behera T.K., Behera S., Bharathi L., John K.J., Simon P.W., Staub J.E. (2010). Bitter gourd: Botany, horticulture, breeding. Hortic. Rev..

[3] Dandawate P.R., Subramaniam D., Padhye S.B., Anant S. (2016). Bitter melon: A panacea for inflammation and cancer. Chin. J. Nat. Med..

[4] Jarvis W., Gubler W., Grove G., Bélanger R.R., Bushnell W.R., Dik A.J., Carver T.L.W. (2002). Epidemiology of powdery mildews in agricultural pathosystems. The Powdery Mildews: A Comprehensive Treatise.

[5] Chen Q., Yu G., Wang X., Meng X., Lv C. (2021). Genetics and resistance mechanism of the cucumber (*Cucumis sativus* L.) against powdery mildew. J. Plant Growth Regul..

[6] Xin M., Wang X., Peng H., Yao Y., Xie C., Han Y., Ni Z., Sun Q. (2012). Transcriptome comparison of susceptible and resistant wheat in response to powdery mildew infection. Genom. Proteom. Bioinform..

[7] Cao Y., Diao Q., Lu S., Zhang Y., Yao D. (2022). Comparative transcriptomic analysis of powdery mildew resistant and susceptible melon inbred lines to identify the genes involved in the response to Podosphaera xanthii infection. Sci. Hortic..

[8] Li W., Deng Y., Ning Y., He Z., Wang G.-L. (2020). Exploiting broad-spectrum disease resistance in crops: From molecular dissection to breeding. Annu. Rev. Plant Biol..

[9] Collum T.D., Culver J.N. (2016). The impact of phytohormones on virus infection and disease. Curr. Opin. Virol..

[10] Wang D., Dawadi B., Qu J., Ye J. (2022). Light-engineering technology for enhancing plant disease resistance. Front. Plant Sci..

[11] Benjamin G., Pandharikar G., Frendo P. (2022). Salicylic acid in plant symbioses: Beyond plant pathogen interactions. Biology.

[12] Fidler J., Graska J., Gietler M., Nykiel M., Prabucka B., Rybarczyk-Płońska A., Muszyńska E., Morkunas I., Labudda M. (2022). PYR/PYL/RCAR receptors play a vital role in the abscisic-acid-dependent responses of plants to external or internal stimuli. Cells.

[13] Ngou B.P.M., Jones J.D., Ding P. (2022). Plant immune networks. Trends Plant Sci..

[14] Yu Y., Gui Y., Li Z., Jiang C., Guo J., Niu D. (2022). Induced systemic resistance for improving plant immunity by beneficial microbes. Plants.

[15] Kachroo A., Kachroo P. (2009). Fatty acid–derived signals in plant defense. Annu. Rev. Phytopathol..

[16] Kouzai Y., Kimura M., Watanabe M., Kusunoki K., Osaka D., Suzuki T., Matsui H., Yamamoto M., Ichinose Y., Toyoda K. (2018). Salicylic acid—Dependent immunity contributes to resistance against Rhizoctonia solani, a necrotrophic fungal agent of sheath blight, in rice and Brachypodium distachyon. New Phytol..

[17] Shine M., Yang J.W., El-Habbak M., Nagyabhyru P., Fu D.Q., Navarre D., Ghabrial S., Kachroo P., Kachroo A. (2016). Cooperative functioning between phenylalanine ammonia lyase and isochorismate synthase activities contributes to salicylic acid biosynthesis in soybean. New Phytol..

[18] Bhuiyan N.H., Selvaraj G., Wei Y., King J. (2009). Gene expression profiling and silencing reveal that monolignol biosynthesis plays a critical role in penetration defence in wheat against powdery mildew invasion. J. Exp. Bot..

[19] Yadav V., Wang Z., Wei C., Amo A., Ahmed B., Yang X., Zhang X. (2020). Phenylpropanoid pathway engineering: An emerging approach towards plant defense. Pathogens.

[20] Rong W., Luo M., Shan T., Wei X., Du L., Xu H., Zhang Z. (2016). A wheat cinnamyl alcohol dehydrogenase TaCAD12 contributes to host resistance to the sharp eyespot disease. Front. Plant Sci..

[21] Yang Q., He Y., Kabahuma M., Chaya T., Kelly A., Borrego E., Bian Y., El Kasmi F., Yang L., Teixeira P. (2017). A gene encoding maize caffeoyl-CoA O-methyltransferase confers quantitative resistance to multiple pathogens. Nat. Genet..

[22] Zipfel C., Felix G. (2005). Plants and animals: A different taste for microbes?. Curr. Opin. Plant Biol..

[23] Jones J.D., Dangl J.L. (2006). The plant immune system. Nature.

[24] Zipfel C. (2008). Pattern-recognition receptors in plant innate immunity. Curr. Opin. Immunol..

[25] Dixon R.A., Achnine L., Kota P., Liu C.J., Reddy M.S., Wang L. (2002). The phenylpropanoid pathway and plant defence—A genomics perspective. Mol. Plant Pathol..

[26] Ma W., Smigel A., Verma R., Berkowitz G.A. (2009). Cyclic nucleotide gated channels and related signaling components in plant innate immunity. Plant Signal. Behav..

[27] He P., Shan L., Sheen J. (2007). Elicitation and suppression of microbe-associated molecular pattern-triggered immunity in plant–microbe interactions. Cell. Microbiol..

[28] Abramovitch R.B., Anderson J.C., Martin G.B. (2006). Bacterial elicitation and evasion of plant innate immunity. Nat. Rev. Mol. Cell Biol..

[29] Geng S., Li A., Tang L., Yin L., Wu L., Lei C., Guo X., Zhang X., Jiang G., Zhai W. (2013). TaCPK2-A, a calcium-dependent protein kinase gene that is required for wheat powdery mildew resistance enhances bacterial blight resistance in transgenic rice. J. Exp. Bot..

[30] Hu Y., Cheng Y., Yu X., Liu J., Yang L., Gao Y., Ke G., Zhou M., Mu B., Xiao S. (2021). Overexpression of two CDPKs from wild Chinese grapevine enhances powdery mildew resistance in Vitis vinifera and Arabidopsis. New Phytol..

[31] Hong K., Gong D., Zhang L., Hu H., Jia Z., Gu H., Song K. (2016). Transcriptome characterization and expression profiles of the related defense genes in postharvest mango fruit against Colletotrichum gloeosporioides. Gene.

[32] Naveed Z.A., Ali G.S. (2018). Comparative transcriptome analysis between a resistant and a susceptible wild tomato accession in response to Phytophthora parasitica. Int. J. Mol. Sci..

[33] Meng H., Sun M., Jiang Z., Liu Y., Sun Y., Liu D., Jiang C., Ren M., Yuan G., Yu W. (2021). Comparative transcriptome analysis reveals resistant and susceptible genes in tobacco cultivars in response to infection by Phytophthora nicotianae. Sci. Rep..

[34] Meng X., Yu Y., Song T., Yu Y., Cui N., Ma Z., Chen L., Fan H. (2022). Transcriptome sequence analysis of the defense responses of resistant and susceptible cucumber strains to Podosphaera xanthii. Front. Plant Sci..

[35] Wang S., Yan W., Yang X., Zhang J., Shi Q. (2021). Comparative methylome reveals regulatory roles of DNA methylation in melon resistance to Podosphaera xanthii. Plant Sci..

[36] Yadav V., Wang Z., Guo Y., Zhang X. (2022). Comparative transcriptome profiling reveals the role of phytohormones and phenylpropanoid pathway in early-stage resistance against powdery mildew in watermelon (*Citrullus lanatus* L.). Front. Plant Sci..

[37] Chen L., Zhang L., Yu D. (2010). Wounding-induced WRKY8 is involved in basal defense in Arabidopsis. Mol. Plant-Microbe Interact. MPMI.

[38] Wasternack C., Song S. (2017). Jasmonates: Biosynthesis, metabolism, and signaling by proteins activating and repressing transcription. J. Exp. Bot..

[39] Bhuvnesh K., Pankaj K., Rajnish S., Arun K. (2021). Regulatory interactions in phytohormone stress signaling implying plants resistance and resilience mechanisms. J. Plant Biochem. Biotechnol..

[40] Bozbuga R. (2020). Expressions of Pathogenesis related 1 (PR1) Gene in Solanum lycopersicum and Influence of Salicylic Acid Exposures on Host-Meloidogyne incognita Interactions. Doklady Biochem. Biophys..

[41] Sood M., Kapoor D., Kumar V., Kalia N., Bhardwaj R., Sidhu G.P.S., Sharma A. (2021). Mechanisms of Plant Defense Under Pathogen Stress: A Review. Curr. Protein Pept. Sci..

[42] Raji M.R., Lotfi M., Tohidfar M., Ramshini H., Sahebani N., Aalifar M., Baratian M., Mercati F., de Michele R., Carimi F. (2022). Multiple fungal diseases resistance induction in Cucumis melo through co-transformation of different pathogenesis related (PR) protein genes. Sci. Hortic..

[43] Knoth C., Eulgem T. (2008). The oomycete response gene LURP1 is required for defense against Hyaloperonospora parasitica in Arabidopsis thaliana. Plant J. Cell Mol. Biol..

[44] Peng J.Y., Huang Y.P. (2005). The signaling pathways of plant defense response and their interaction. Zhi Wu Sheng Li Yu Fen. Zi Sheng Wu Xue Xue Bao = J. Plant Physiol. Mol. Biol..

[45] Ha C.M., Rao X., Saxena G., Dixon R.A. (2021). Growth-defense trade-offs and yield loss in plants with engineered cell walls. New Phytol..

[46] Xie M., Zhang J., Tschaplinski T.J., Tuskan G.A., Chen J.G., Muchero W. (2018). Regulation of Lignin Biosynthesis and Its Role in Growth-Defense Tradeoffs. Front. Plant Sci..

[47] Cardoni M., Gómez-Lama Cabanás C., Valverde-Corredor A., Villar R., Mercado-Blanco J. (2022). Unveiling Differences in Root Defense Mechanisms Between Tolerant and Susceptible Olive Cultivars to Verticillium dahliae. Front. Plant Sci..

[48] Gallego-Giraldo L., Posé S., Pattathil S., Peralta A.G., Hahn M.G., Ayre B.G., Sunuwar J., Hernandez J., Patel M., Shah J. (2018). Elicitors and defense gene induction in plants with altered lignin compositions. New Phytol..

[49] Sattler S.E., Funnell-Harris D.L. (2013). Modifying lignin to improve bioenergy feedstocks: Strengthening the barrier against pathogens?. Front. Plant Sci..

[50] Bhuiyan N.H., Liu W., Liu G., Selvaraj G., Wei Y., King J. (2007). Transcriptional regulation of genes involved in the pathways of biosynthesis and supply of methyl units in response to powdery mildew attack and abiotic stresses in wheat. Plant Mol. Biol..

[51] Bhuiyan N.H., Selvaraj G., Wei Y., King J. (2009). Role of lignification in plant defense. Plant Signal. Behav..

[52] Zierold U., Scholz U., Schweizer P. (2005). Transcriptome analysis of mlo-mediated resistance in the epidermis of barley. Mol. Plant Pathol..

[53] Junxin Z., Xihuan Y., Tiran H., Huan L., Fang L., Meixia Y., MingFeng Y., Lanqing M. (2022). Overexpressing 4-coumaroyl-CoA ligase and stilbene synthase fusion genes in red raspberry plants leads to resveratrol accumulation and improved resistance against Botrytis cinerea. J. Plant Biochem. Biotechnol..

[54] Xiang C., Liu J., Ma L., Yang M. (2020). Overexpressing codon-adapted fusion proteins of 4-coumaroyl-CoA ligase (4CL) and stilbene synthase (STS) for resveratrol production in Chlamydomonas reinhardtii. J. Appl. Phycol..

[55] Chang J., Guo Y., Yan J., Zhang Z., Yuan L., Wei C., Zhang Y., Ma J., Yang J., Zhang X. (2021). The role of watermelon caffeic acid O-methyltransferase (ClCOMT1) in melatonin biosynthesis and abiotic stress tolerance. Hortic. Res..

[56] Tronchet M., Balagué C., Kroj T., Jouanin L., Roby D. (2010). Cinnamyl alcohol dehydrogenases-C and D, key enzymes in lignin biosynthesis, play an essential role in disease resistance in Arabidopsis. Mol. Plant Pathol..

[57] Kawasaki T., Koita H., Nakatsubo T., Hasegawa K., Wakabayashi K., Takahashi H., Umemura K., Umezawa T., Shimamoto K. (2006). Cinnamoyl-CoA reductase, a key enzyme in lignin biosynthesis, is an effector of small GTPase Rac in defense signaling in rice. Proc. Natl. Acad. Sci. USA.

[58] Almagro L., Gómez Ros L.V., Belchi-Navarro S., Bru R., Ros Barceló A., Pedreño M.A. (2009). Class III peroxidases in plant defence reactions. J. Exp. Bot..

[59] Garcia-Brugger A., Lamotte O., Vandelle E., Bourque S., Lecourieux D., Poinssot B., Wendehenne D., Pugin A. (2006). Early signaling events induced by elicitors of plant defenses. Mol. Plant-Microbe Interact. MPMI.

[60] Zhao J., Davis L.C., Verpoorte R. (2005). Elicitor signal transduction leading to production of plant secondary metabolites. Biotechnol. Adv..

[61] Cao Y., Diao Q., Chen Y., Jin H., Zhang Y., Zhang H. (2020). Development of KASP Markers and Identification of a QTL Underlying Powdery Mildew Resistance in Melon (*Cucumis melo* L.) by Bulked Segregant Analysis and RNA-Seq. Front. Plant Sci..

[62] Liu G., Sheng X., Greenshields D.L., Ogieglo A., Kaminskyj S., Selvaraj G., Wei Y. (2005). Profiling of wheat class III peroxidase genes derived from powdery mildew-attacked epidermis reveals distinct sequence-associated expression patterns. Mol. Plant-Microbe Interact. MPMI.

[63] Chen O., Deng L., Ruan C., Yi L., Zeng K. (2021). Pichia galeiformis Induces Resistance in Postharvest Citrus by Activating the Phenylpropanoid Biosynthesis Pathway. J. Agric. Food Chem..

[64] Zhu Q., Gao P., Wan Y., Cui H., Fan C., Liu S., Luan F. (2018). Comparative transcriptome profiling of genes and pathways related to resistance against powdery mildew in two contrasting melon genotypes. Sci. Hortic..

[65] Xu L., Zhu L., Tu L., Liu L., Yuan D., Jin L., Long L., Zhang X. (2011). Lignin metabolism has a central role in the resistance of cotton to the wilt fungus Verticillium dahliae as revealed by RNA-Seq-dependent transcriptional analysis and histochemistry. J. Exp. Bot..

[66] Lenardon M.D., Munro C.A., Gow N.A. (2010). Chitin synthesis and fungal pathogenesis. Curr. Opin. Microbiol..

[67] Moeder W., Urquhart W., Ung H., Yoshioka K. (2011). The role of cyclic nucleotide-gated ion channels in plant immunity. Mol. Plant.

[68] Ali R., Ma W., Lemtiri-Chlieh F., Tsaltas D., Leng Q., von Bodman S., Berkowitz G.A. (2007). Death don’t have no mercy and neither does calcium: Arabidopsis CYCLIC NUCLEOTIDE GATED CHANNEL2 and innate immunity. Plant Cell.

[69] Leba L.J., Cheval C., Ortiz-Martín I., Ranty B., Beuzón C.R., Galaud J.P., Aldon D. (2012). CML9, an Arabidopsis calmodulin-like protein, contributes to plant innate immunity through a flagellin-dependent signalling pathway. Plant J. Cell Mol. Biol..

[70] Reddy A.S., Ali G.S., Celesnik H., Day I.S. (2011). Coping with stresses: Roles of calcium- and calcium/calmodulin-regulated gene expression. Plant Cell.

[71] Boudsocq M., Sheen J. (2013). CDPKs in immune and stress signaling. Trends Plant Sci..

[72] Romeis T., Herde M. (2014). From local to global: CDPKs in systemic defense signaling upon microbial and herbivore attack. Curr. Opin. Plant Biol..

[73] Pandey S.P., Somssich I.E. (2009). The role of WRKY transcription factors in plant immunity. Plant Physiol..

[74] Wang H., Gong W., Wang Y., Ma Q. (2023). Contribution of a WRKY Transcription Factor, ShWRKY81, to Powdery Mildew Resistance in Wild Tomato. Int. J. Mol. Sci..

[75] Xu X., Wang R., Chao J., Lin Y.E., Jin Q., He X., Luo S., Wu T. (2015). The expression patterns of *Cucumis sativus* WRKY (CsWRKY) family under the condition of inoculation with Phytophthora melonis in disease resistant and susceptible cucumber cultivars. Can. J. Plant Sci..

[76] Xiao S., Brown S., Patrick E., Brearley C., Turner J.G. (2003). Enhanced transcription of the Arabidopsis disease resistance genes RPW8. 1 and RPW8. 2 via a salicylic acid–dependent amplification circuit is required for hypersensitive cell death. Plant Cell.

[77] Sakata Y., Kubo N., Morishita M., Kitadani E., Sugiyama M., Hirai M. (2006). QTL analysis of powdery mildew resistance in cucumber (*Cucumis sativus* L.). Theor. Appl. Genet..

[78] Wang N., Wang R., Wang R., Chen S. (2018). Transcriptomics analysis revealing candidate networks and genes for the body size sexual dimorphism of Chinese tongue sole (*Cynoglossus semilaevis*). Funct. Integr. Genom..

